# Providing baseline data for conservation–Heart rate monitoring in captive scimitar-horned oryx

**DOI:** 10.3389/fphys.2023.1079008

**Published:** 2023-02-24

**Authors:** Peter Leimgruber, Nucharin Songsasen, Jared A. Stabach, Megan Horning, Dolores Reed, Tara Buk, Arielle Harwood, Lawrence Layman, Christopher Mathews, Morgan Vance, Paul Marinari, Kelly E. Helmick, Kristina M. Delaski, Lisa H. Ware, Julia C. Jones, Jose L. P. Silva, Timothy G. Laske, Rosana Nogueira Moraes

**Affiliations:** ^1^ Conservation Ecology Center, Smithsonian National Zoo & Conservation Biology Institute, Front Royal, VA, United States; ^2^ Center for Species Survival, Smithsonian National Zoo & Conservation Biology Institute, Front Royal, VA, United States; ^3^ Department of Conservation Medicine, Smithsonian National Zoo & Conservation Biology Institute, Front Royal, VA, United States; ^4^ Department of Statistics, Federal University of Parana, Curitiba, Paraná, Brazil; ^5^ Department of Surgery, University of Minnesota, Minneapolis, MN, United States; ^6^ Cardiac Ablation Solutions, Medtronic Inc., Mounds View, MN, United States; ^7^ Department of Physiology, Federal University of Parana, Curitiba, Paraná, Brazil

**Keywords:** biologging, physiologging, heart rate, conservation physiology, activity, circadian rhythms, *Oryx dammah*, desert ungulates

## Abstract

Heart rate biologging has been successfully used to study wildlife responses to natural and human-caused stressors (e.g., hunting, landscape of fear). Although rarely deployed to inform conservation, heart rate biologging may be particularly valuable for assessing success in wildlife reintroductions. We conducted a case study for testing and validating the use of subcutaneous heart rate monitors in eight captive scimitar-horned oryx (*Oryx dammah*), a once-extinct species that is currently being restored to the wild. We evaluated biologger safety and accuracy while collecting long-term baseline data and assessing factors explaining variation in heart rate. None of the biologgers were rejected after implantation, with successful data capture for 16–21 months. Heart rate detection accuracy was high (83%–99%) for six of the individuals with left lateral placement of the biologgers. We excluded data from two individuals with a right lateral placement because accuracies were below 60%. Average heart rate for the six scimitar-horned oryx was 60.3 ± 12.7 bpm, and varied by about 12 bpm between individuals, with a minimum of 31 bpm and a maximum of 188 bpm across individuals. Scimitar-horned oryx displayed distinct circadian rhythms in heart rate and activity. Heart rate and activity were low early in the morning and peaked near dusk. Circadian rhythm in heart rate and activity were relatively unchanged across season, but hourly averages for heart rate and activity were higher in spring and summer, respectively. Variation in hourly heart rate averages was best explained by a combination of activity, hour, astronomical season, ambient temperature, and an interaction term for hour and season. Increases in activity appeared to result in the largest changes in heart rate. We concluded that biologgers are safe and accurate and can be deployed in free-ranging and reintroduced scimitar-horned oryx. In addition to current monitoring practices of reintroduced scimitar-horned oryx, the resulting biologging data could significantly aid in 1) evaluating care and management action prior to release, 2) characterizing different animal personalities and how these might affect reintroduction outcomes for individual animals, and 3) identifying stressors after release to determine their timing, duration, and impact on released animals. Heart rate monitoring in released scimitar-horned oryx may also aid in advancing our knowledge about how desert ungulates adapt to extreme environmental variation in their habitats (e.g., heat, drought).

## Introduction

Anthropogenic activities now alter and influence nearly every biogeochemical process on our planet and have resulted in climate change, declines in extent and function of ecosystems, and species extinction (IPBES, 2019). Conservation physiology is a relatively new discipline focusing on studying and quantifying physiological responses of organisms to anthropogenic change (Wikelski and Cooke, 2006; Cooke et al., 2020). Essentially, conservation physiology is measuring, monitoring, and counteracting how human-caused changes in the environment affect the wellbeing, welfare, fitness, and survival of organisms. Measuring physiological parameters in free-ranging animals is a logistical and methodological challenge and is increasingly relying on biologging (Hawkes et al., 2021). Because biologging technologies are often new and relatively untested, there is a need to develop baseline data under controlled conditions and validate tools prior to broad application (Madliger et al., 2018). Here, we present a case study for testing and validating the use of heart rate monitors in scimitar-horned oryx (*Oryx dammah*), a once-extinct species that is currently being restored to the wild (Chuven et al., 2018).

Biologging has become an important tool for answering critical questions in animal ecology, physiology, behavior, and conservation (Cooke, 2008; Kays et al., 2015; Wilson et al., 2015; Hawkes et al., 2021; Williams et al., 2021). This increasing use of biologging has been facilitated by technological advances in sensor development (e.g., battery life, miniaturization of devices, transmission technology), which have produced smaller, longer-lasting devices with greater storage capacity (Kays et al., 2015; Williams et al., 2021). Most of the resulting biologging research is centered on tracking wildlife (i.e., logging animal locations), measuring body position and acceleration along major axes, and registering ambient environmental variables (e.g., temperature, salinity). Physiologging, a subfield of biologging focused on recording of animal physiological states (e.g., temperature, heart rate) using implanted or attached electronic devices, has also accelerated in laboratory and captive settings, but its application in free-living species is more limited because of additional technological challenges (Hawkes et al., 2021; Williams et al., 2021).

Heart rate monitoring has been the workhorse of physiologging (Hawkes et al., 2021). Initial research in wildlife species began in the early 1970s on unrestrained but captive animals (Cherkovich and Tatoyan, 1973; Moen, 1978) and by the late 1970s proceeded to studies on free-ranging wildlife using longer-range biotelemetry systems (Kanwisher et al., 1978; MacArthur et al., 1979; MacArthur et al., 1982). This pioneering work highlighted the potential for linking heart rate to metabolic rates (Moen, 1978), behavior (Kanwisher et al., 1978), as well as human-induced stress (MacArthur et al., 1979; MacArthur et al., 1982). While older devices had serious technological constraints (i.e., size and weight of the system, transmission), requiring highly invasive surgical procedures and significantly limiting data acquisition and quality, new generations of heart rate monitors have overcome some of these challenges. As a consequence, heart rate monitoring has been effectively used for quantifying changes in metabolic rate with season (e.g., hibernation, hypometabolism) (Arnold et al., 2004; Turbill et al., 2011; Evans et al., 2016; Græsli et al., 2020b), determining energy expenditure during different types of behaviors (Theil et al., 2004; Zizkova et al., 2013), assessing the effects of social dominance (Wascher et al., 2008; Turbill et al., 2013), quantifying how animals respond to natural or human-caused stressors (e.g., hunting, landscape of fear) (Laske et al., 2011; Ditmer et al., 2015a; Ditmer et al., 2015b; Støen et al., 2015; Græsli et al., 2020a), assessing stress (Pohlin et al., 2017), and monitoring animal welfare (Jukan et al., 2017). Despite these advances, applications of heart rate monitoring to inform wildlife management and conservation, especially for threatened and endangered species, are still rare (Pohlin et al., 2017; Moraes et al., 2021).

Physiologging may be especially useful for monitoring success in wildlife reintroductions and translocations, an area that is considered challenging and has often been lacking rigorous study (Berger-Tal et al., 2020). Biologging of heart rate can provide information about how an animal responds to novel environments and particularly to natural (e.g., drought, heat) and human-caused stressors (e.g., poaching livestock, proximity to humans). However, prior to the deployment of heart rate monitors in released and free-ranging animals, they should be tested for function, usefulness, and accuracy in captive populations. Captive animals in zoos and wildlife parks represent key source populations for efforts to restore species once extinct in the wild (Conde et al., 2011; Chuven et al., 2018). In addition, the management of captive populations allows for controlled research to establish baseline data on the biology and ecology of endangered species.

The scimitar-horned oryx represents an excellent case study example, with a large global herd of captive individuals providing the source population for recent reintroductions (Chuven et al., 2018; Ogden et al., 2020). The species once roamed across the Sahara-Sahelian region of northern Africa but went extinct in the 1980s (Newby, 1978; Harris et al., 2009) because of overhunting, poaching, and habitat decline due to overgrazing and desertification (Newby, 1988). The species disappeared from the wild before being systematically studied and there are only a few very limited observations that give information about the ecology and behavior of species in the wild. Thus, little is known about its habitat requirements. Nevertheless, the species has recently been reintroduced to the Ouadi Rimé-Ouadi Achim Faunal Reserve (OROA) in Chad (Chuven et al., 2018) and continued monitoring of the released population *via* satellite-GPS tracking and field observation provides new and important data on the species’ ecology and habitat requirements (Mertes et al., 2019; Majaliwa et al., 2022). This ongoing reintroduction has resulted in over 350 animals roaming freely in the wild (Mertes, pers. Comm.). Heart rate monitoring before, during and after the release of these animals would likely provide novel and important information on the effects of management on individual animals and their ability to adjust.

We conducted a case study to investigate the application and use of subcutaneous biologgers in captive scimitar-horned oryx. In our research, we had four objectives:

1. Determine whether subcutaneous heart rate monitors can be safely used in scimitar-horned oryx.
2. Evaluate if the heart rate monitors provide accurate data for the species studied.
3. Collect long-term baseline data on heart rate in scimitar-horned oryx.
4. Explore the effects of animal activity, hour, ambient temperature, daylight length and astronomical season on heart rate.

The results of our research provide solid support for implementing biologging of heart rate in free-ranging scimitar-horned oryx. We discuss our findings within the context of previous work in ungulates and highlight potential applications of heart rate monitoring specifically for the reintroduction of scimitar-horned oryx and in a broader context of monitoring individual performance during translocation and reintroduction.

## Materials and methods

### Study subjects

**FIGURE 1:**
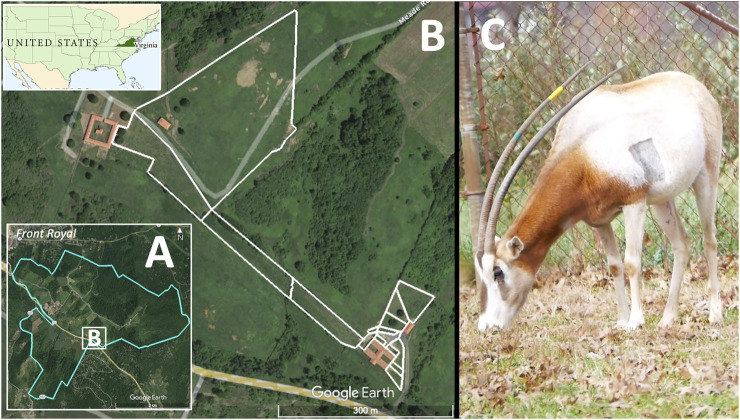
Study site location and study species. The Smithsonian Conservation Biology Institute (SCBI; 1,400 ha) **(A)** is located outside the town of Front Royal, VA, United States, in the foothills of the Appalachian Mountains. A series of enclosures covering 7.47 ha **(B)** were used to manage a herd of scimitar-horned oryx, including the eight individuals for this study. Animals were provided access to fenced pasture (white delimiting lines) as well as shelters and barns. The right panel **(C)** shows a female scimitar-horned oryx grazing inside a fenced pasture at SCBI 6 days after being implanted with a heart monitor. The shaved rectangular spot in the left parasternal position marked the site where the biologger was injected subcutaneously.

The Smithsonian Conservation Biology Institute (SCBI) manages a captive herd of scimitar-horned oryx to facilitate breeding, scientific research and contribute to the *in situ* and *ex situ* conservation of the species. SCBI is a 1,440-ha facility for research and conservation of endangered species and their ecosystems and is located outside the town of Front Royal, VA, in the foothills of the Appalachian Mountains (Figures 1A, B). The scimitar-horned oryx herd at SCBI is maintained in several enclosures over an area of 7.47 ha (Figure 1B). Animals are moved between fenced pastures with free access to grazing and to shelters or barn facilities that protect animals from the weather and enable veterinary and animal care procedures. We selected eight adult scimitar-horned oryx (Figure 1C) for this study: one vasectomized male and seven non-pregnant females (Table 1). Initially, the studied animals were part of a social group of 18 adult females, five offspring of the year and the vasectomized male (“Sweeny”). After about 6 months, the male was permanently separated from the female herd and transferred to his own pasture and barn, in visual and olfactory proximity to other oryx. Animal care personnel interacted twice daily with scimitar-horned oryx for feeding, check-ups, care, and cleaning of the barn at approximately 08h00 and 14h00 local time. The animals had free access to pastures and hay and received pellet food once a day at about 15h00. Between August and November 2019, the female herd was temporarily kept in the largest grazing area (outlined by white lines in Figure 1B) due to barn maintenance.

**TABLE 1:** Demographic information for scimitar-horned oryx implanted with a biologger (Reveal LINQ™, Medtronic, MN, United States) for heart rate monitoring at the Smithsonian Conservation Biology Institute, Front Royal, VA, United States.

Subject	Sex	Age (years)	Body weight (kg)	Biologger placement^a^	Monitoring time (days)
Savannah	F	1.9	112	left	574
Scout	F	5.5	145	left	518
Sweeny	M	7.4	158	left	607
Loretta	F	13.5	129	left	656
Chari	F	7.4	135	left	581
Bamako	F	7.5	116	left	658
Rose	F	2.7	119	right	571
Glenda	F	2.4	117	right	571

^a^ To assess whether placement affected heart rate monitoring accuracy, we place biologgers subcutaneously either in a left or right parasternal position.

### Heart rate monitor implantation and data collection

We relied on the Reveal LINQ™ (Medtronic, Minneapolis, MN) biologgers equipped with the latest version of the custom software “B-Ware” (Laske et al., 2018) for heart rate monitoring. Several studies have successfully used identical or similar biologgers for heart rate monitoring in other mammalian species (Laske et al., 2021; Moraes et al., 2021). The device (4.0 mm × 7.2 mm x 44.8 mm; mass 2.4 g, volume 1.2 cc) is designed for subcutaneous positioning close to the heart (Laske et al., 2018). It has a long lifespan (up to 3 years), large storage capacity (three years for daily averages and up to 400 days for high density parameters added in B-Ware), and wireless capacity for remote web-based monitoring (Laske et al., 2018).

For the biologger placement and programming, we followed previously published procedures (Moraes et al., 2021), with some changes in anatomical location and detection parameters to account for unique cardiac anatomy and physiology in bovids that affect how electrocardiograms (ECGs) are generated (Santamarina et al., 2001; Tiusanen and Pastell, 2016). In bovids, the combination of a high voltage ventricular repolarization wave (T) and a long ST interval (time between the end of ventricular depolarization and beginning of ventricular repolarization) may cause dual heartbeat detection by automated algorithms due to T wave detection as ventricular depolarization (Santamarina et al., 2001; Tiusanen and Pastell, 2016).

Scimitar-horned oryx were anesthetized with a combination of 1.3–1.5 mg/kg of ketamine (ZooPharm, Laramie WY, 82070 United States) and 0.015–0.025 mL/kg of a commercial premix of butorphanol, azaperone, and medetomidine (BAM ™, ZooPharm, Laramie WY, 82070 United States) administered by intramuscular injection (IM). Naltrexone and atipamezole (ZooPharm, Laramie WY, 82070 United States) were administered IM at 2:1 butorphanol and 5:1 medetomidine, respectively, as reversal agents. Using aseptic techniques, we inserted the device subcutaneously in a left parasternal location over the heart area (∼45° on the sagittal plane) in six animals (Figure 1C) and tested a right parasternal placement in two females (named “Rose” and “Glenda”) (Table 1). We verified optimal positioning by monitoring the R-wave amplitude (>0.15 mV). To minimize double counting of each heartbeat, with R-waves being detected as ventricular senses, we adjusted the blank time (minimum time interval between ventricular senses) between 150 and 300 ms, as needed. The incision site was closed with simple interrupted sutures (2–0 absorbable monofilament poliglecaprone 25 or braided polyglactin 910 suture) in the subcutaneous and skin layers. To determine whether these subcutaneous biologgers can be used safely in scimitar-horned oryx, animal care staff visually inspected individuals twice daily to ensure we would detect and treat possible reactions (e.g., redness, swelling) at the location of the implant.

Animals were implanted between November 2018 and January 2020 and monitored on average for 592 days (Table 1). Data collection followed methods detailed for maned wolves (*Chrysocyon brachyurus*) in Moraes et al. (2021). We programmed the biologgers to continuously record 2-min average heart rate in beats per minute (bpm). In addition, the biologger recorded the ECGs from the last 10 episodes of tachycardia (heart rate ≥125 bpm) or asystole (at least 4.5 s between heartbeats). To measure activity, we relied on the biologger’s internal triaxial accelerometer to record the number of minutes that the animal was active (=movement exceeded the speed of ∼1,600 m h^−1^) at 15 min intervals (Medtronic, Minneapolis, MN).

To download the different data types stored by the biologger, we used remote transmission and direct telemetry. For remote transmissions, we installed a monitor (MyCareLink^®^, Medtronic) inside the barn, about 3 m from the animals’ usual resting place. The biologger was programmed to connect wirelessly to its respective monitor every 2 h and transmit a 10-s ECG in real time and the last 10 ECGs associated with any episode of tachycardia or asystole. ECG data were transmitted *via* the Global System for Mobile Communications (GSM) to the individual repository created for each animal on the Medtronic CareLink^®^ network. Because full data downloads can only be done through direct telemetry, we conducted opportunistic direct downloads during veterinary checks or management procedures that required physical restraint of the animals in a hydraulic Tamer^®^ (Fauna Research, Red Hook, New York). During the direct telemetry download, data were accessed by holding the telemetry head (CareLink^®^ model 2090 programmer) near the implant site (approximately 5 cm telemetry range). To improve the accuracy of ventricular detection, we also used the first data download opportunity (∼a month after biologger placement) to adjust the blank time interval of all eight biologgers to 300 ms.

### Evaluating accuracy of HR measurements

To evaluate the accuracy of the biologger heartbeat detection, we analyzed 883 10-s ECGs automatically sent to the GSM network. We compared the heart rate calculated from the time intervals between R-waves (RR intervals) by the biologger with the RR intervals calculated using the *findpeaks* function (pracma package in R; Borchers, 2022); and ECG visual confirmation. Biologger detection was considered accurate when the absolute difference between the biologger heart rate (observed heart rate) and the validated heart rate (expected heart rate) was <10%.

### Data processing

Although the internal clocks of the biologgers were synchronized to their location at the time of placement, the time recordings exhibited slight offsets when verified during data download. To assure the accuracy of the recorded time, we used linear regressions on individual datasets according to Moraes et al. (2021). The combined data from the eight oryx were then filtered to exclude the 2-min averages obtained with blank intervals below 300 ms and heart rate values below 30 bpm (minimum value confirmed by ECG). To calculate individual and overall heart rate averages and ranges, we used the 2-min heart rate average dataset. For the analysis of factors influencing heart rate, we summarized 2-min heart rate averages into hourly intervals. For activity, the 15-min intervals data were summed to calculate the total number of minutes per hour (total hourly activity) and per day (total daily activity). In addition, as a proxy for resting heart rate, we summarized heart rate for the hourly intervals when animals were inactive (i.e., total activity = 0).

To assess the role of ambient temperature in influencing average heart rate we downloaded 30-min interval Single Aspirated Air Temperature data provided by the National Ecological Observatory Network (National Ecological Observatory Network (NEON), 2021) for SCBI, the core site for the Mid-Atlantic region of NEON. For our study, we averaged these data at hourly intervals.

To test the influence of day length on heart rate, we relied on the *daylength* function provided by the geosphere package in R (Hijmans et al., 2021). This function uses the latitude of the study location (SCBI; Lat: 38.89292) and date to compute the photoperiod. We also tested the influence of astronomical seasons on heart rate, with each season starting on the respective dates of the solstice and equinox in the Northern hemisphere.

### Statistical analysis

Initial temporal plots of hourly heart rate and activity confirmed circadian and seasonal waveform patterns that are best approximated by polynomial functions. We used Generalized Additive Models for Location, Scale and Shape (GAMLSS; Rigby et al., 2005) to model the influence of animal activity, hour, ambient temperature, day length, and astronomical season on heart rate in scimitar-horned oryx. GAMLSS provides univariate models in which heart rate can be modeled as an additive function of multiple explanatory variables, allowing for the use of cubic splines for analyzing waveform data (Rigby et al., 2005). We converted the temperature from Celsius to Fahrenheit to avoid negative values and facilitate analysis with cubic splines. To account for repeated measurements and individual variation of heart rate, we included individuals as random effects. To determine which covariates were most important, we compared competing models with varying variable combinations and selected the best fit model based on its Akaike Information Criterion (AIC) value (Table 4).

We also checked whether autocorrelation resulted in an overestimation of partial fixed effects of our selected model. First, we used the *acf* function in R to determine the autocorrelation structure of the residuals of our null model (*hrm0* in Table 4). We found strong autocorrelation coefficients for lag 1 (*acf* = 0.71) and lag 24 (acf = 0.55) (Supplementary Figure S1), confirming the daily rhythms observed in the hourly heart rate plots. Next, we fit a model with the same structure as our selected model (*hrm8* in Table 4) but using a random subset of only 500 data points per animal and compared the results with the model fit with the full dataset (67,230 data points). Fitting the model with the randomly selected small dataset resulted in lower residual autocorrelation coefficients of lag 1 (*acf =* 0.34) and lag 24 (*acf =* 0.05) (Supplementary Figure S4). Yet, the outputs for the two models generated nearly identical values for the coefficient estimates, standard errors, *t* and P values (Supplementary Table S3). Thus, we confirmed that autocorrelation did not change the relative importance of covariates in influencing heart rate, as assessed by our selected model using the complete dataset.

All data processing and statistical analysis were performed in R (R Core Team, 2021) and RStudio (RStudio Team, 2020).

## Results

We successfully implanted eight scimitar-horned oryx with heart rate monitors. None of the biologgers were rejected after implantation and all devices collected data for 16–21 months (Table 1), confirming that the monitors can be safely used in scimitar-horned oryx.

**FIGURE 2:**
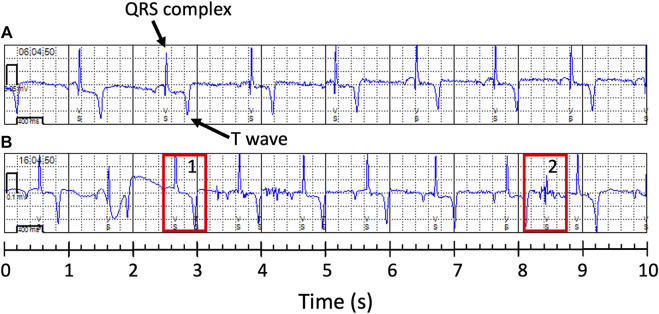
Examples of 10-s electrocardiogram (ECG) obtained from two female scimitar-horned-oryx with a heart monitor. Note that the ventricular sense (VS) marks correctly indicate the QRS complexes (arrow) in the accurate ECG in **(A)** (female “Bamako”), but not in **(B)** (female “Rose”). In **(B)**, the biologger was detecting T waves (rectangle 1) and electric noise (rectangle 2) as ventricular senses, increasing the number of ventricular senses to 14 instead of 10.

**FIGURE 3:**
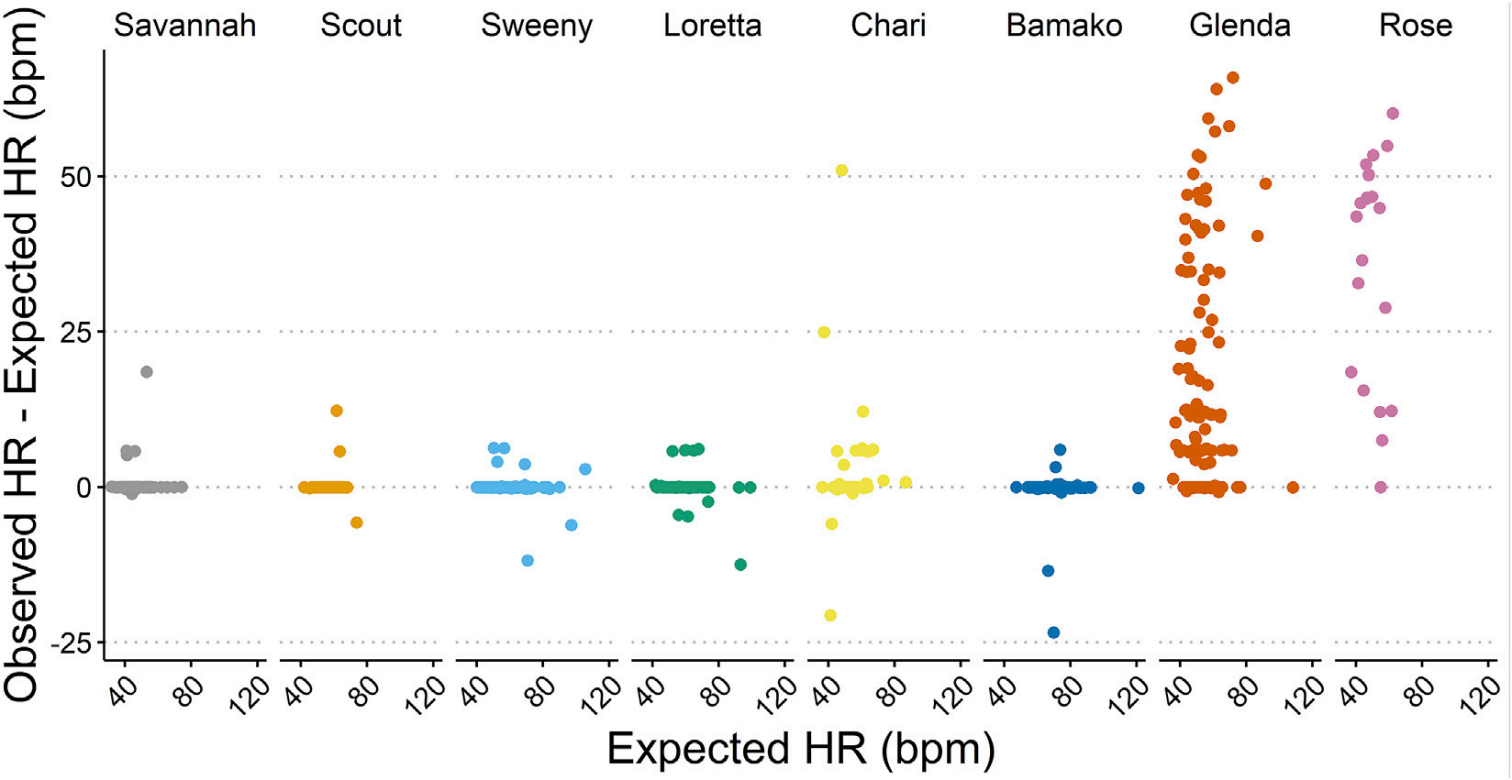
Accuracy of the heart rate (HR) measurements based on the absolute difference (bpm) between observed HR (detected by the biologger) and expected HR (confirmed by ECG validation).

Accuracy assessments showed that the two biologgers with a right lateral placement were oversensing (Table 2; Figures 2B, 3), resulting in data accuracies below 60%. All data from these two animals were excluded from further data analysis to avoid biasing results. For the ECG of the six scimitar oryx implanted on the left side (*n* = 647), the detection accuracy was between 83% and 99%. The validated and recorded heart rate differed by less than 5% in 94% of ECGs and less than 10% in 96% of ECGs. The adjusted R-squared between recorded and validated heart rate was 0.92. Setting the blank interval to 300 ms increased accuracy. For example, the proportion of accurate ECGs for the female “Loretta” increased from 67% to 98%, when obtained using the respective blank intervals of 150 ms (*n* = 42) or 300 ms (*n* = 108).

**TABLE 2:** Accuracy of heart rate averages based on comparison of RR intervals from biologger data with RR intervals calculated from 10-s ECG strips.

Oryx	ECGs validated (n)	Accurate ECGs (n)	Accuracy (%)
Savannah	70	66	94
Scout	64	63	98
Sweeny	276	273	99
Loretta	108	106	98
Chari	46	38	83
Bamako	83	81	98
Glenda^a^	164	84	51
Rose^a^	22	1	5
Total	833	712	85

^a^ Animals with biologgers in the right parasternal position, resulting in oversensing and lower accuracies.

Average heart rate across for the six scimitar-horned oryx was 60.3 ± 12.7 bpm (Table 3), and varied by about 12 bpm between individuals, with “Savannah” having the lowest and “Bamako” the highest averages. The lowest 2-min average heart rate observed in our study was 31 bpm and the highest 188 bpm (“Sweeny”) (Table 3). Visual inspection of the ECG strips revealed that heart rates could drop as low as 30 and increase as high as 212 bpm, as recorded for “Savannah” right before being physically restrained in a Tamer^®^ for an intervention. The average heart rate was 10.5 bpm lower when the animals were inactive (57.0 ± 10.9 bpm) compared with when they were active (67.5 ± 11.0 bpm).

**TABLE 3:** Heart rate (HR) averages (bpm) for six captive scimitar-horned oryx. HR averages were continuously recorded at 2-min intervals for an average of 592 days per individual. Data for two study animals were removed because right sternal placement of the monitor reduced data accuracy.

Subject	Sex	HR Average	
		Mean ± SD	Range
Savannah	F	53.3 ± 11.0	31–150
Scout	F	56.7 ± 10.6	31–150
Sweeny	M	59.5 ± 11.4	36–188
Loretta	F	60.5 ± 11.9	32–162
Chari	F	63.3 ± 15.3	31–154
Bamako	F	66.7 ± 11.6	34–150
Total		60.3 ± 12.7	31–188

^a^ Heart rate averages were continuously obtained at 2-min intervals.

**FIGURE 4:**
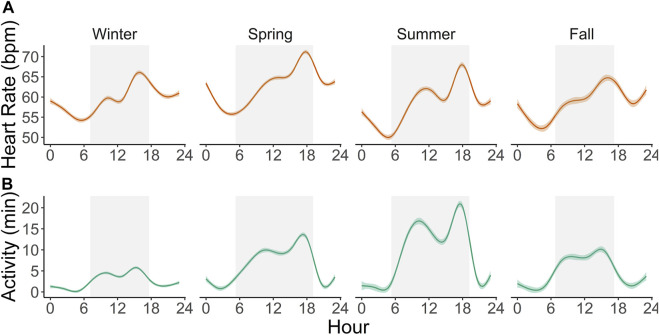
Circadian profile of hourly heart rate averages **(A)** and active minutes per hour **(B)** in six captive scimitar-horned oryx continuously monitored with an implantable biologger for an average of 592 days. Solid colored lines represent smoothing curves fitted with the function *stat_smooth (method = “gam”)* in R and color shaded areas represent 95% confidence intervals. Gray rectangles represent the average duration of the day (from dawn to dusk) for each astronomical season. Similar patterns are observed throughout each season, with noticeable biphasic increases in activity and heart rate and maximum peaks occurring before dusk.

**FIGURE 5:**
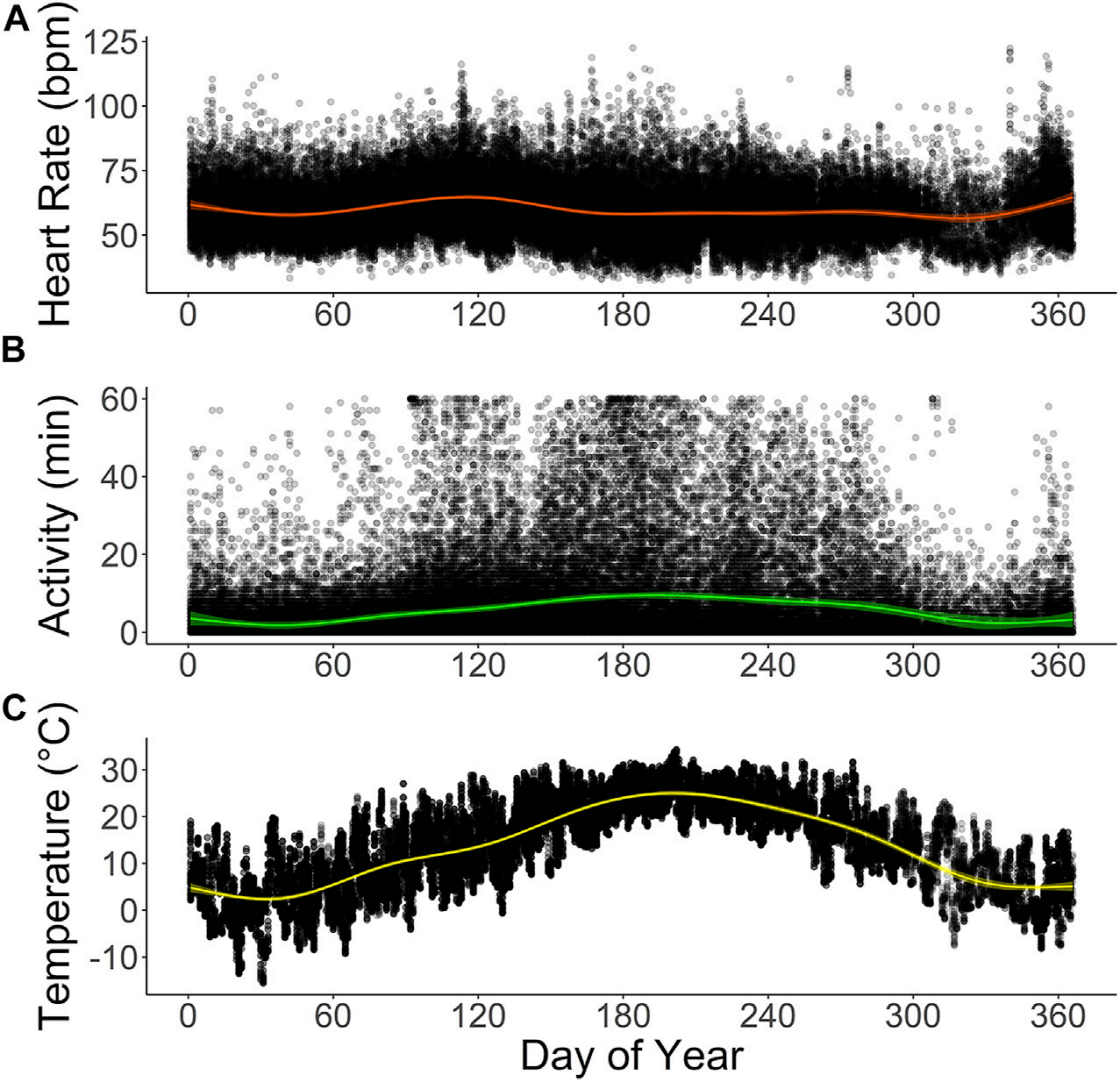
Annual profiles of **(A)** hourly averages of heart rate (bpm) and **(B)** active minutes in six captive scimitar-horned oryx. Animals were implanted with a subcutaneous biologger that continuously recorded heart rate as 2-min averages and active minutes (animal’s movement exceeded the speed of ∼1,600 m h^−1^) at 15 minute-intervals. Hourly averages for ambient temperature (°C) at the study site are provided in **(C)**. Colored lines show smoothing curves fitted with the function *stat_smooth (method = “gam”)* in R and colored shaded areas represent 95% confidence intervals. Black dots represent raw data points for each variable. The time on the X-axes corresponds to the dates of the Julian calendar for the study period.

Scimitar-horned oryx displayed a distinct circadian rhythm in the heart rate, which was relatively constant over the seasons (Figure 4A), despite a significant interaction of season on the partial contribution of hour on heart rate averages (Table 5; Supplementary Figure S3). The lowest hourly heart rate averages were recorded from 05h00–07h00. Heart rate averages then steadily increased to a 24-h maximum around dusk. Daily peaks changed slightly between seasons, with later peaks in summer and earlier peaks in winter, following changes in day length. Summer had a more pronounced daily rhythm, with an increase of 17 bpm in average heart rate between morning and evening twilight. In winter, the difference was only 14 bpm. An annual profile for hourly heart rate averages is shown in Figure 5A.

The total daily activity of the captive scimitar-horned oryx was low and varied depending on the season. During the winter, the animals were very inactive during the day and at night, with an average daily activity of only 60 ± 57 min. In the spring, the animals became more active during the day, increasing the total daily average to 151 ± 119 min, peaking in the summer at 214 ± 144 min. By the fall, total daily activity averages fell to 107 ± 89 min. We also observed distinct circadian and seasonal patterns for hourly averages of activity (Figures 4B, 5B). The number of minutes of activity per hour increased between early morning and dusk, and then decreased quickly at night (Figure 4B). These trends were more pronounced in summer, with peaks in activity around 17h00 to 18h00 (Figure 4B). Throughout the year, hourly averages of activity tend to follow increases in hourly averages for ambient temperature (Figures 5B, C).

**FIGURE 6:**
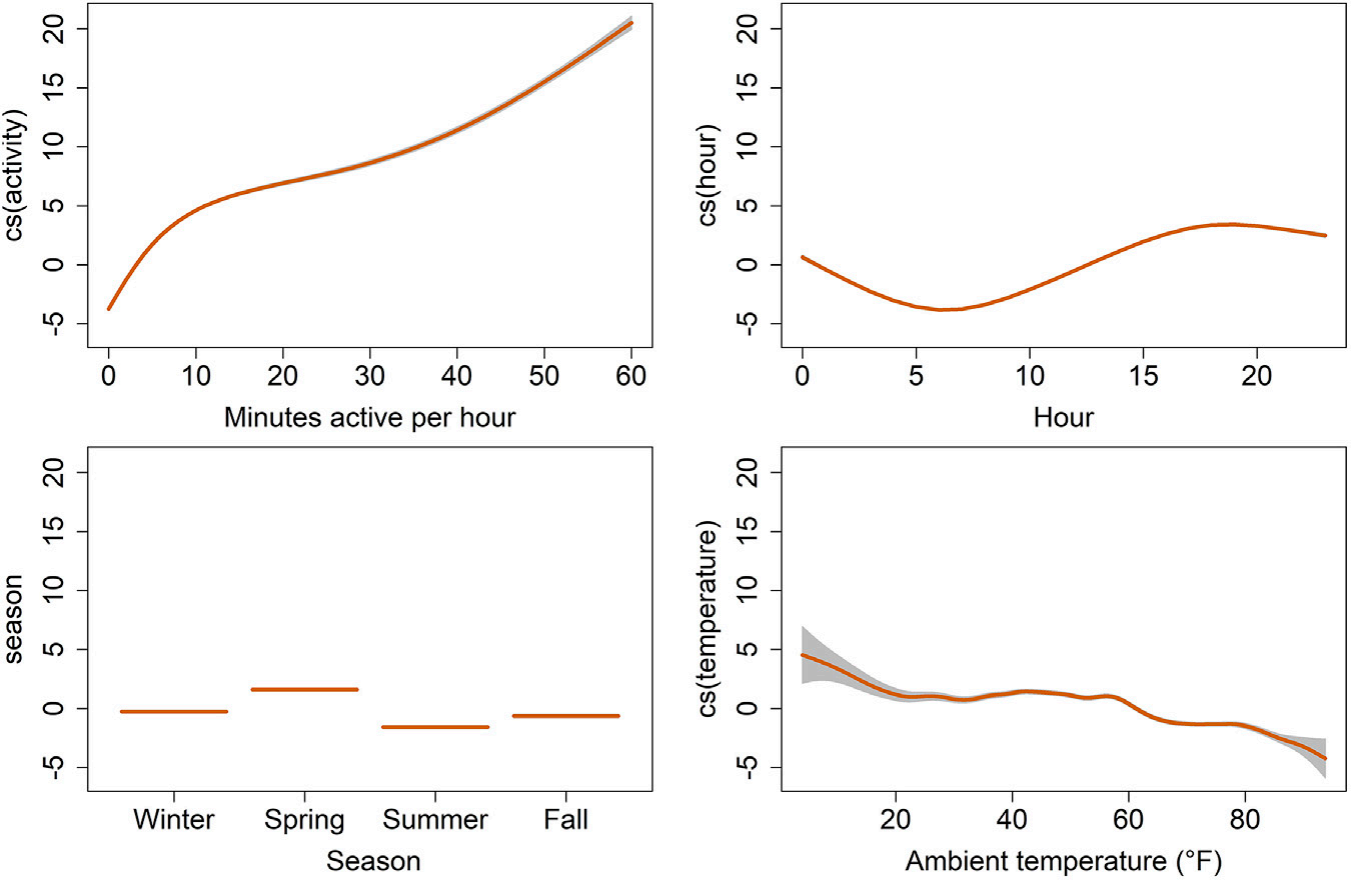
Termplot of the best fit generalized additive model for location, scale, and shape (GAMLSS), showing on the y-axis the partial effects of selected explanatory variables on the hourly average of heart rate (bpm) for six captive scimitar-horned oryx. Heart rate estimates are represented by the trend lines [cubic spline (cs) smooth predictors for activity, hour and ambient temperature and linear predictors for season]. The gray shaded areas correspond to the estimates’ standard errors. Individual scimitar-horned oryx were included as random effects and individual variation in heart rate was confirmed in a null model.

We tested and compared multiple explanatory models to assess which combination of factors best explained variation in heart rate (Table 4). Our best performing model (i.e, lowest AIC, hrm8) showed that variation in hourly heart rate average was best explained by a combination of activity, hour, astronomical season, ambient temperature, and an interaction term for hour and season (Table 5; Figure 6; Supplementary Figure S3). Increases in activity appeared to result in the greatest changes in heart rate, followed by hour, as can be seen by the model terms in Figure 6. GAMLSS analysis also revealed a seasonal influence with higher heart rate during the winter and spring and lower heart rate during the summer and fall (Figure 6). Temperature was overall less important but increased heart rate at very low temperatures (below ∼20°F/-7°C) and decreased heart rate at very high temperatures (above ∼80°F/27°C) (Figure 6). While the interaction term was significant, we found only slight differences for the importance of hour in explaining heart rate between seasons and the relative daily pattern was unchanged (Supplementary Figure S3). Finally, there was considerable individual variation in heart rate (Table 3) which was accounted for using animal id as a random effect.

**TABLE 4:** The Akaike information criterion (AIC) was used to determine the best fit of Generalized Additive Models for Location, Scale, and Shape (GAMLSS) for assessing which factors most affect heart rate in six captive scimitar-horned oryx. Lower AIC represent a better performance.

Model	Variables^a^	Df	AIC	dAIC
hrm8	intercept + cs (activity) + cs (hour)*season + cs (temperature) + re (random = ∼1|individual)	38	455319	0
hrm7	intercept + cs (activity) + cs (hour) + season + cs (temperature) + re (random = ∼1|individual)	35	455429	410
hrm6	intercept + cs (activity) + cs (hour) + season +	20	456447	1128
	re (random = ∼1|individual)			
hrm5	intercept + cs (activity) + cs (hour) + cs (temperature) +	32	457201	1882
	re (random = ∼1|individual)			
hrm4	intercept + cs (activity) + cs (hour) +	17	460780	5461
	re (random = ∼1|individual)			
hrm3	intercept + cs (activity) + cs (temperature) + cs (day length) +	32	466373	11054
	re (random = ∼1|individual)			
hrm2	intercept + cs (activity) + cs (temperature)+	28	467428	12109
	re (random = ∼1|individual)			
hrm1	intercept + cs (activity)+	13	469656	14337
	re (random = ∼1|individual)			
hrm0	intercept + re (random = ∼1|individual)	9	490877	35558

^a^ Cs, cubic spline; re, random effect.

**TABLE 5:** Summary of the best-fit model using GAMLSS and family distribution Johnson’s SU (*JSU*) to assess the importance of covariates for hourly heart rate average (bpm) in six captive scimitar-horned oryx. A generalized *R* ^*2*^ value of 51.5 was calculated using the *Rsq* function and non-linear effects were fit using cubic spline (*cs*) function of the gamlss R package.

Variable	Estimate	Std. Error	t	P
Intercept	57.21	0.1113	499.48	<0.001
cs (Activity)	0.43	0.0026	162.59	<0.001
cs (Hour)	0.26	0.0039	83.15	<0.001
Spring	0.72	0.0819	22.80	<0.001
Summer	−1.81	0.1096	−12.09	<0.001
Fall	−1.14	0.0978	−3.83	<0.001
cs (Ambient temperature)	−0.05	0.0025	−21.02	<0.001
cs (Hour): Spring	0.10	0.0094	10.79	<0.001
cs (Hour): Summer	0.05	0.0101	4.70	<0.001
cs (Hour): Fall	0.07	0.0129	5.25	<0.001

## Discussion

Our study provides the first record of continuous heart rate values for scimitar-horned oryx collected over an extended period, providing valuable baseline data for comparisons with future studies in this species and ecologically similar desert ungulates. Using an implantable biologger, we were able to accurately monitor heart rate and animal activity and identify factors that best explain variation in heart rate. Deployment of these biologgers in free-ranging and released scimitar-horned oryx would significantly advance our ability to better understand their ecology and habitat requirements in the wild. It would also enhance the ability of reintroduction managers to evaluate how management before, during, and after release will affect the fitness of individual animals.

### Biologger use and accuracy

The devices used in our study required minimally invasive surgery for subcutaneous insertion and proved to be safe for use in scimitar-horned oryx. Not a single device was rejected or caused an infection. This is consistent with other research that utilized similar biologgers in brown bear (*Ursus arctos*; Støen et al., 2015; Evans et al., 2016), American black bear (*Ursus americanu*s; Ditmer et al., 2015a; Laske et al., 2018), maned wolf (Moraes et al., 2021), moose (*Alces alces;* Græsli et al., 2020a), and that are currently being tested for as many as nine other species (Laske et al., 2021).

Accuracy assessments are rarely provided in current research on heart rate, though there is a high potential for biasing estimates upwards during high activity or stress periods because of oversensing. Depending on the cardiac electrophysiology of the study species (e.g., high or low average heart rate at rest relative to other species), muscle noise (i.e., electromyographic activity from skeletal muscle) and/or electric noise stemming from movement of the device or the surrounding subcutaneous tissue can reduce the performance of a device’s automatic detection algorithm which was optimized for human clinical use (Laske et al., 2021). Such issues are often addressed through data filtering. But without adequate reference data filtering may become a challenge.

Our results demonstrated that the biologgers were reliable in providing continuous data and the data was highly accurate, depending on the anatomical location of the implant and the detection settings used. We evaluated accuracy by calculating RR intervals from the ECG strip and comparing them with simultaneous RR intervals recorded by the device’s automatic detection algorithm. Similar accuracy assessment techniques have previously been used to investigate heart rate in Svalbard reindeer (*Rangifer tarandus platyrhynchus*) (Trondrud et al., 2021). Accuracies in our study were very similar to what they reported for their highest quality data, with 94% and 96% (our study) vs. 96% and 98.5% (Trondrud et al., 2021), for data deviating by less than 5% or 10% from ECG validated data, respectively. Similarly, we found minor differences in adjusted R-squared between recorded and validated heart rate in our study (0.92) and theirs (0.96).

We tested the placement of the biologgers in left and right parasternal positions and varying blank time intervals, trying to prevent dual counting of ventricular depolarizations by the device’s algorithm. Placement on the left parasternal location and setting the blanking period after a ventricular sense at 300 ms reduced oversensing and provided highly accurate data. Implanting the biologger on the right side did not improve heartbeat detection. Data from two animals had to be excluded from the analysis because of a higher rate of erroneous detection of T-waves and electric noise as heartbeats (Figure 2B). Due to a reduced number of individuals tested for right side placement, we cannot properly assess the source of the electrical noise, but the biologger could potentially be detecting electrical signals originating from the different compartments of the stomach (Wierzbicka et al., 2021). Erroneous automated detection of ventricular depolarization (QRS) may limit long-term monitoring of heart rate in other ungulate and cetacean species, which share similar anatomical and electrophysiological characteristics of the heart (Ono et al., 2009; De Almeida et al., 2015). In ungulates, Purkinje fibers, a major component of the mammalian ventricular electrical conduction and propagation system, form subendocardial and intramural networks that extend excitation across the ventricular walls. The morphology and function of the Purkinje cell network are reflected in the ECG electrical axis of the heart in ungulates (Detweiler, 2010) and can lead to larger and longer T waves, with relatively smaller QRS complexes as seen in sheep (Kviesulaitis et al., 2018) and pig (Hamlin, 2010). When using implantable biologgers (Græsli et al., 2020a) or sensors attached to the skin (Kviesulaitis et al., 2018), T waves might be detected by the algorithm as ventricular depolarization (QRS complex), leading to double counting of heartbeats.

### Baseline heart rate data in scimitar-horned oryx

Accurate reference values are essential for improving care (e.g., informing anesthesia protocols) and management of captive and wild scimitar-horned oryx. To our knowledge, our study is the first to report on physiological heart rate ranges in adult scimitar-horned oryx or other desert-adapted bovids. Previously reported values for anaesthetized desert bovids varied depending on the anesthetic protocol used, with values slightly above or below our overall average (60 bpm). Drug-induced stimulant or depressant effects in the heart have been observed in scimitar-horned oryx (Pearce and Kock, 1989), Arabian oryx (Ancrenaz et al., 1996), and addax (Portas et al., 2003). We also observed low heart rates (41 ± 11 bpm) during the procedure to implant the biologger, suggesting a cardiovascular depressant effect of the anesthetic protocol used in our study.

Despite differences in body size and experimental conditions, the range in heart rate observed for scimitar-horned oryx in our study is similar to data obtained by telemetry from unrestrained individuals of other bovid species, including the relatively small roe deer (*Capreolus capreolus*; Theil et al., 2004; Reimoser, 2012; Arnold, 2020) and the largest member of the deer family, the moose (Græsli et al., 2020b) (Supplementary Table S4). Our results were also similar to those reported for red deer (*Cervus elaphus*; Arnold et al., 2004; Turbill et al., 2011; Reimoser, 2012; Arnold, 2020), eland (*Taurotragus oryx*; Zizkova et al., 2013) and reindeer (*Rangifer tarandus*; Mesteig et al., 2000; Trondrud et al., 2021) (Supplementary Table S4). Heart rate monitors also have proved useful in detecting episodes of tachycardia (heart rate >125 bpm) in scimitar-horned oryx and relating these episodes to specific environmental challenges or events. For example, one animal (“Savannah”) exhibited a heart rate of 212 bpm while confined in a Tamer^®^ prior to a care procedure, which is similar to values recorded for roe deer (Theil et al., 2004) when trotting (184 bpm) or fleeing from humans (255 bpm) and moose when chased by dogs (195 bpm; Græsli et al., 2020a). Our findings are also consistent with those from a previous study on maned wolves, where tachycardia events were linked to confinement, social interactions, or other exogenous challenges (Moraes et al., 2021). Similarly, heart rate monitors were used to detect extremely low or high heart rates in wild black bears and brown bears (Laske et al., 2018).

### Factors influencing heart rate in scimitar-horned oryx

Long-term heart rate patterns in mammals are closely tied to changes in metabolic rate as the result of seasonal variation in ambient temperature (Arnold et al., 2004; Arnold et al., 2006; Arnold, 2020), activity (Theil et al., 2004; Græsli et al., 2020b), food availability and nutrition (Arnold et al., 2004; Turbill et al., 2011; Arnold et al., 2018; Arnold, 2020), and reproduction (Arnold et al., 2004; Wascher et al., 2018; Ruf et al., 2021). These patterns are often overlaid, and sometimes masked, by physiological responses to other stressors, mainly intra- and inter-species interactions (e.g., dominance hierarchies, competition, predator avoidance; Chabot et al., 1996; Turbill et al., 2013), extreme environmental events (e.g., hard winters, heat; Das et al., 2016; Arnold et al., 2018), and human-caused disturbance (e.g., roads, hunting; Laske et al., 2011; Ditmer et al., 2015a; Ditmer et al., 2015b; Støen et al., 2015; Græsli et al., 2020a).

In our study, heart rate was most strongly influenced by activity, hour, season, and to a lesser extent by ambient temperature as can be seen in our results from GAMLSS (Figure 6). Daily rhythms of activity and heart rate were both characterized by increasing values during the day towards a peak near dusk, followed by decreasing values with low activity and heart rate at night, reaching the lowest values early in the morning (Figure 4). Because food availability was not a constraint, daily activity and heart rate patterns were mostly related to animal care, feeding routines, and weather conditions. Animals would lie inactive most of the night in the barn, moving out to the pastures around 08h00, during the barn cleaning routine. Outside foraging time was minimal in winter, with the herd spending most of the time inside the barn under heat sources. In summer, animals sought shelter only at midday due to the heat load and to avoid insects. In addition to locomotor activity, we believe food intake and ruminal activity, which affect the annual heart rate in deer (Mesteig et al., 2000; Turbill et al., 2011), may play a role in the daily heart rate fluctuations in the scimitar-horned oryx in our study, possibly due to combined changes in cardiac output, blood flow in the gastrointestinal tract, ruminal temperature and metabolic rate. These factors, however, remain to be tested in scimitar-horned oryx.

The seasonal heart rate in the captive scimitar-horned oryx was impacted by factors other than animal activity. Although scimitar-horned oryx were less active in winter, their heart rate was higher in winter than in summer (Figures 4, 5). This finding is in contradiction with what has been described for free-living wild northern ungulates, which tend to reduce their metabolism and heart rate over the winter in response to extreme cold (Arnold, 2020). Yet, this hypometabolic response to extreme cold can be prevented when the diet is unrestricted (Turbill et al., 2011), which could be the case in our study. As has already been observed in domesticated ruminants (Young, 1983; Brosh, 2007), the thermal stress caused by low ambient temperatures in winter may have activated thermoregulatory mechanisms to increase the metabolic rate and heat production of scimitar-horned oryx, resulting in increases in resting heart rate. Our results show that the overall average of hourly heart rate when scimitar-horned oryx were inactive was five bpm higher in winter (56.7 bpm) than in summer (51.9 bpm) and the partial cubic spline values for ambient temperature in our best fit model (Figure 6) suggest that scimitar-horned oryx elevate their heart rate at very low temperatures. In addition, we believe that psychological stress in winter, as a result of multiple animals sharing a heat source inside a confined shelter, should have impacted heart rate as well. Lack of space to avoid agonistic behaviors has long been recognized as a source for stress in captive animals (Morgan and Tromborg, 2006; Moraes et al., 2021) and research on fecal corticoid metabolites in captive scimitar-horned oryx herds also reported increased corticoid levels during winter months (Pauling et al., 2017), which were attributed to confined housing in the winter.

### Potential of heart rate monitoring for scimitar-horned oryx reintroduction

Biologging of heart rate may be especially valuable for use in reintroduction projects where animals are exposed to a wide range of stressors stemming from changes in care, management, and environment. Animals respond to these stressors through complex interactions between the brain and the heart that continuously modulate autonomous heart control and adrenal gland activity. These responses can be measured indirectly by quantifying glucocorticoids that accumulate over time in urine, feces, and hair (Heimbürge et al., 2019; Palme, 2019), or more directly by continuously recording heart rate. The latter provides high temporal resolution and facilitates the detection and timing of specific stressors. As animals adjust to new management conditions or environments, we expect peaks in heart rate and a reduction in heart rate variability due to a temporary increase in cardiac adrenergic tone and catecholamines blood levels. The magnitude and duration of the heart responses to stressors, would vary among individuals depending on the context and the animal’s personality and previous experiences.

Deploying heart rate monitors prior to the release should be relatively straightforward. During this stage, the animals are mostly under human care, which allows the implantation of monitors and regular downloads. Heart rate monitors would enable evaluation of different care and management actions and would be particularly effective in monitoring and reducing stress during transport (e.g., mode of transport, use of anesthetics, types of crates), acclimation (e.g., duration of acclimation period, social grouping), and release (e.g., timing, soft vs. hard release).

In combination with behavioral observations and experimentation prior to release, heart rate monitoring could also be used to categorize animal personalities, which have been shown to affect survival during reintroduction (Bremner-Harrison et al., 2004; Sinn et al., 2014; Germano et al., 2017). Several studies have linked heart rate and heart rate variability to individual’s behavioral traits in animals (Kovács et al., 2015; Finkemeier et al., 2019). Defining personality types for scimitar-horned oryx prior to release and then using satellite-GPS tracking could inform future selection of animals for release with a goal of maximizing reintroduction success. Existing research provides evidence that this may be feasible, because scimitar-horned oryx behavior is influenced by past experiences (i.e., management background) and social context (Mertes et al., 2019; Majaliwa et al., 2022; Mertes et al., 2022). In addition, we found considerable individual variation in heart rate patterns in our study (Supplementary Table S1).

The use of the biologgers in scimitar-horned oryx post-release is logistically challenging but possible. Ideally, individual animals would be implanted with a biologger and equipped with a satellite-GPS collar before to being shipped from their source population to the release site. Currently, released individuals are being recaptured after one or two years to replace the tracking collar (Mertes, pers. Comm), which could allow opportunistic data downloads from the heart rate monitors. In the near future, we hope to be able to send biologger data *via* Bluetooth to the satellite-GPS transmitter for upload to an internet server. When integrated with GPS tracking, post-release heart rate may be valuable for identifying the location, timing, and duration of stressors, and their short- and long-term impacts on the animal. Examples of assessing such stressors already exist for other species. Subcutaneous heart rate monitors have been used to evaluate stress in wildlife from exposure to unmanned aerial vehicles (Ditmer et al., 2015b), roads (Ditmer et al., 2018), and hunting (Støen et al., 2015). Continued biologging over longer time periods can be used to determine important thresholds for management, such as the minimum buffer distance from roads or human settlements. It can also provide an objective metric for how long it takes for an animal to adjust and habituate to new conditions.

Scimitar-horned oryx are among the most threatened ungulates globally (Newby, 1988; Devillers and Devillers-Terschuren, 2006; IUCN SSC Antelope Specialist Group, 2016; Woodfine and Gilbert, 2016). Although the restoration of a wild population appears to be successful, the species will require continued monitoring and conservation support. Heart rate biologging may prove to be a valuable tool in these restoration efforts to assess and improve conservation and reintroduction management, and to advance our knowledge about the ecological requirements of the species.

## Data Availability

The datasets presented in this study can be found online at figshare: https://doi.org/10.25573/data.21391068.v3.
