# Supplementary material for: Providing baseline data for conservation–Heart rate monitoring in captive scimitar-horned oryx
Source: Front Physiol. 2023 Feb 24;14:1079008. doi: 10.3389/fphys.2023.1079008 (PMC9998487; doi:10.3389/fphys.2023.1079008)
Supplement: Supplementary file 1 [file Table1.DOCX]

**Providing Baseline Data for Conservation–Heart Rate Monitoring in Captive Scimitar-horned Oryx**

Supplementary Material

**TABLE S1**. Summary of our null gamlss model (*hrm0*) fit with the full dataset (67320 data points), ordered by subject and time and subjects as random effects.

******************************************************************************

Family: c("JSU", "Johnson SU")

Call:gamlss(formula = HRmean ~ 1 + re(random = ~1 | subject), family = JSU(), data = dados)

Fitting method: RS()

---------------------------------------------------------------------------------------------------------------------

Mu link function: identity

Mu Coefficients:

Estimate Std. Error t value Pr(>|t|)

(Intercept) 60.06425 0.03667 1638 <2e-16 ***

Signif. codes: 0 ‘***’ 0.001 ‘**’ 0.01 ‘*’ 0.05 ‘.’ 0.1 ‘ ’ 1

---------------------------------------------------------------------------------------------------------------------

NOTE: Additive smoothing terms exist in the formulas:

i) Std. Error for smoothers are for the linear effect only.

ii) Std. Error for the linear terms maybe are not accurate.

---------------------------------------------------------------------------------------------------------------------

No. of observations in the fit: 67320

Degrees of Freedom for the fit: 8.998256

Residual Deg. of Freedom: 67311

at cycle: 35

Global Deviance: 490858.9

AIC: 490876.9

SBC: 490958.9

******************************************************************************

Random coefficients$subject

(Intercept)

“Savannah” -6.3032340

“Scout” -3.0213933

“Sweeny” -0.3843595

“Loretta” 0.3988858

“Chari” 2.6009115

“Bamako” 6.7091895

******************************************************************************


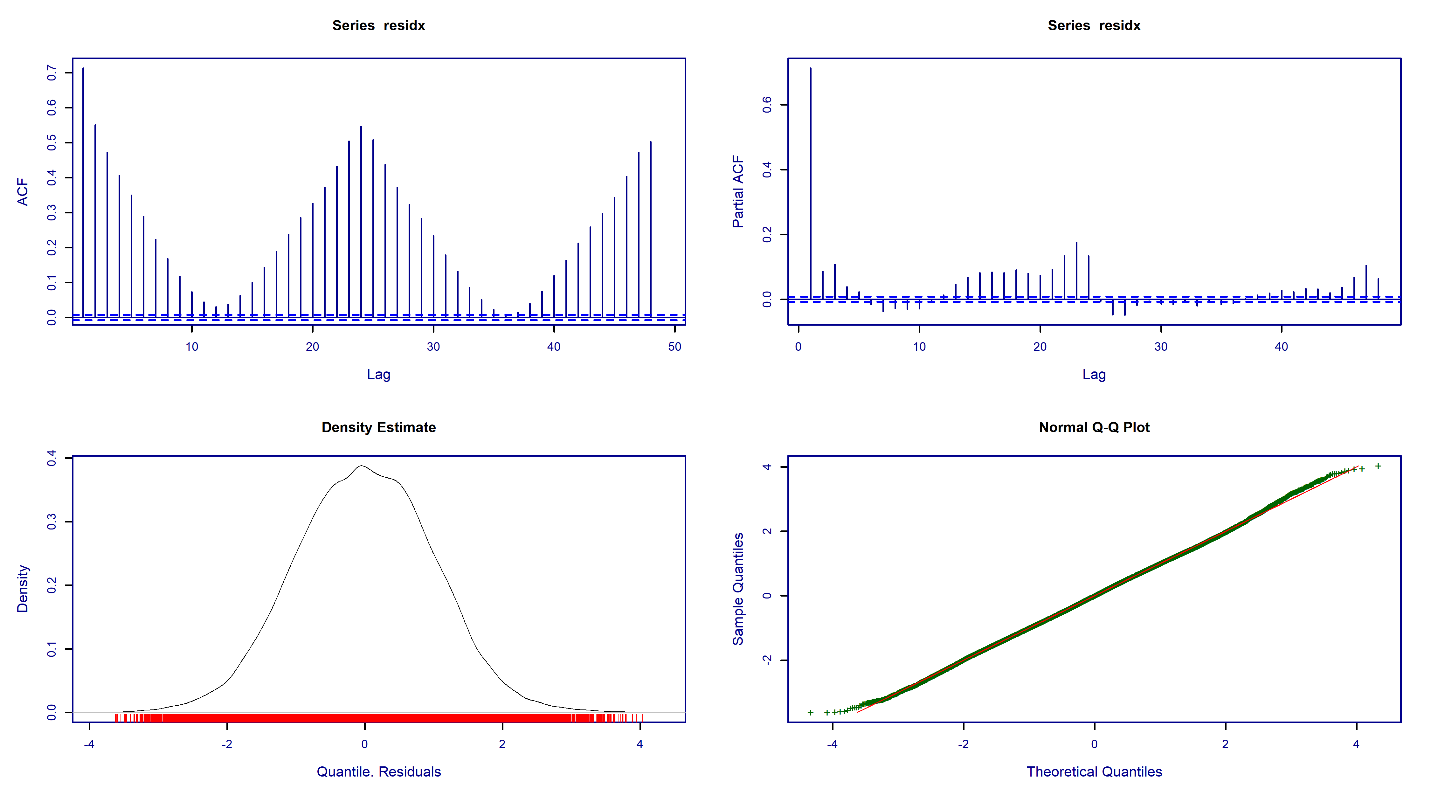


**FIGURE S1**. Diagnostic plots of our null model (*hrm0*) using the full dataset (67320 data points), ordered by subject and time. ACF plots show strong autocorrelation of the data and circadian seasonality, with autocorrelation coefficients of lag 1 = 0.71 and lag 24 = 0.55.

**TABLE S2**. Summary of our selected model (*hrm8*) fit with the full dataset and subjects as random effects. Animal activity, hour, astronomical season, and ambient temperature were included as covariates.

******************************************************************************

Family: c("JSU", "Johnson SU")

Call: gamlss(formula = HRmean ~ cs(activity) + cs(hour) * astron.season + cs(Temperature) +

re(random = ~1 | subj.num), family = JSU, data = na.omit(dados)

Fitting method: RS()

------------------------------------------------------------------

Mu link function: identity

Mu Coefficients:

Estimate Std. Erro t value Pr(>|t|)

(Intercept) 57.215774 0.130904 437.082 < 2e-16 ***

cs(activity) 0.425702 0.002630 161.845 < 2e-16 ***

cs(hour) 0.267685 0.006826 39.216 < 2e-16 ***

astron.seasonSpring 0.720386 0.133749 5.386 7.22e-08 ***

astron.seasonSummer -1.813715 0.157801 -11.494 < 2e-16 ***

astron.seasonFall -1.144126 0.176141 -6.496 8.33e-11 ***

cs(Temperature) -0.053406 0.002488 -21.464 < 2e-16 ***

cs(hour):astron.seasonSpring 0.101869 0.009444 10.786 < 2e-16 ***

cs(hour):astron.seasonSummer 0.047700 0.010147 4.701 2.59e-06 ***

cs(hour):astron.seasonFall 0.067892 0.012919 5.255 1.48e-07 ***

Signif. codes: 0 ‘***’ 0.001 ‘**’ 0.01 ‘*’ 0.05 ‘.’ 0.1 ‘ ’ 1

------------------------------------------------------------------

Sigma link function: log

Sigma Coefficients:

Estimate Std. Error t value Pr(>|t|)

(Intercept) 1.999151 0.003854 518.8 <2e-16 ***

---

Signif. codes: 0 ‘***’ 0.001 ‘**’ 0.01 ‘*’ 0.05 ‘.’ 0.1 ‘ ’ 1

---------------------------------------------------------------------------------------------------------------------

NOTE: Additive smoothing terms exist in the formulas:

i) Std. Error for smoothers are for the linear effect only.

ii) Std. Error for the linear terms maybe are not accurate.

---------------------------------------------------------------------------------------------------------------------

No. of observations in the fit: 67320

Degrees of Freedom for the fit: 38.27091

Residual Deg. of Freedom: 67281.73

at cycle: 25

Global Deviance: 455242.9

AIC: 455319.5

SBC: 455668.4

******************************************************************************


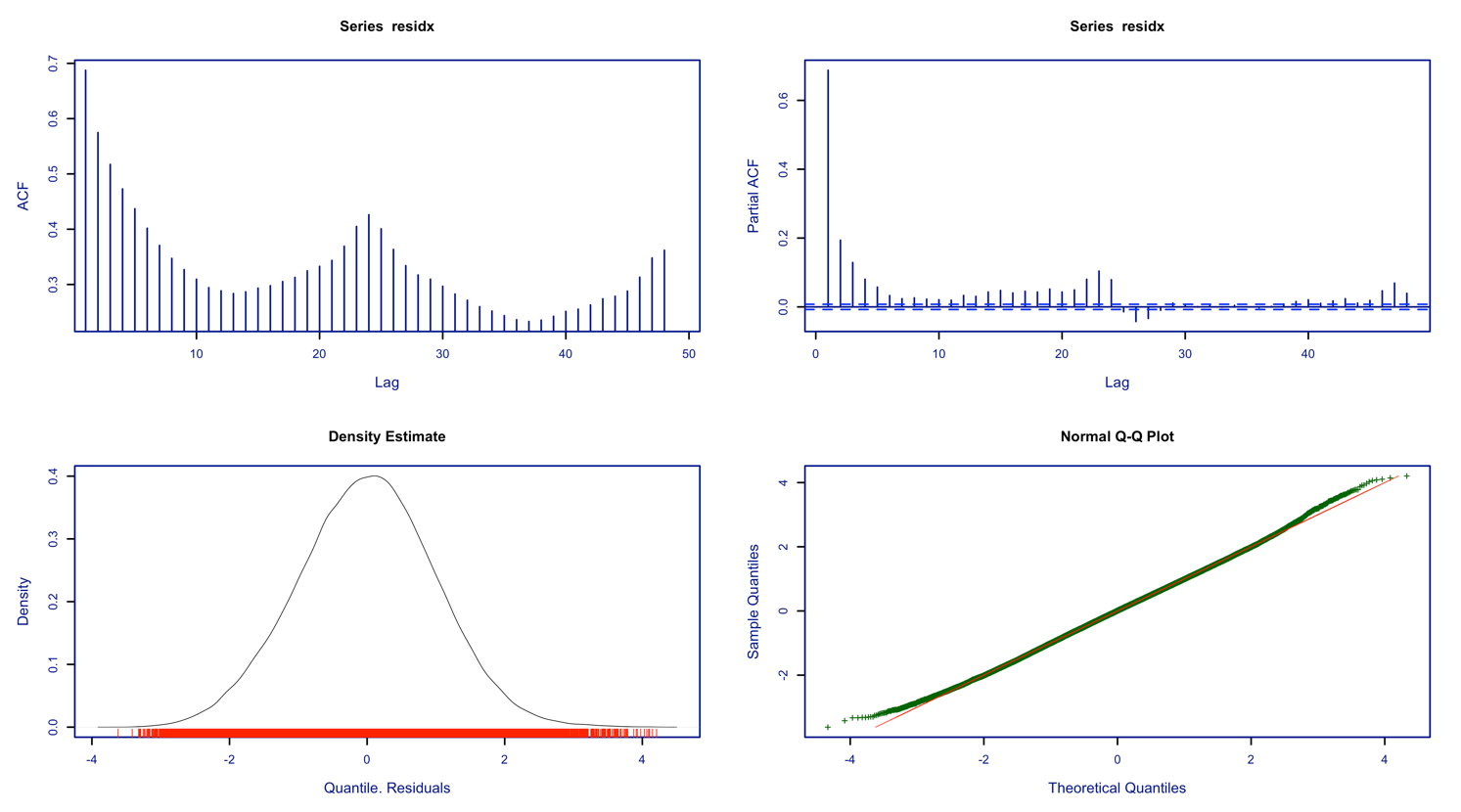


**FIGURE S2**. Diagnostic plots of our best fit model (*hrm8*) using the full dataset (67320 data points), ordered by subject and time. ACF plots show strong autocorrelation of the data and circadian seasonality, with autocorrelation coefficients of lag 1 = 0.69 and lag 24 = 0.43.


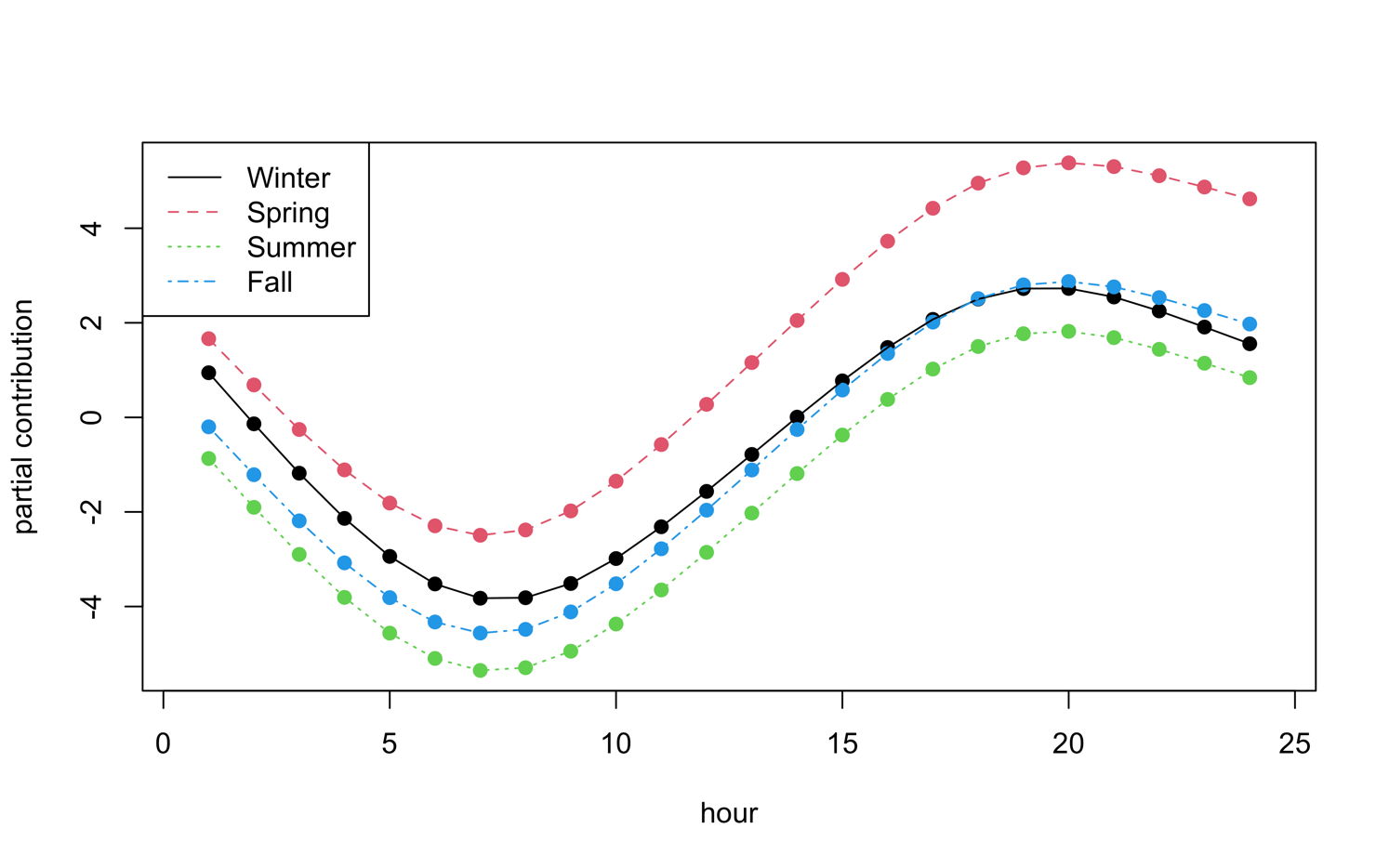


**FIGURE S3**. Plots of the interaction terms for our best fit model (*hrm8; hour*season*) using the full dataset (67320 data points), showing seasonal differences for the partial contribution of hour on heart rate averages (bpm). Plot created by using the *plot2way* function from GAMLSS package in R.

**TABLE S3**. Summary of a gamlss model fit with a random sample of 500 data points per subject (*hrm8.random*). Subjects were included as random effects and animal activity, hour, astronomical season, and ambient temperature as covariates. The formula is the same as the full model (*hrm8*) and we confirmed that reducing the autocorrelation coefficients of the residuals (by randomly selecting 3000 points out of 67320) did not significantly change model estimates and P values.

******************************************************************************

Family: c("JSU", "Johnson SU")

Call: gamlss(formula = HRmean ~ cs(activity) + cs(hour) + astron.season + cs(Temperature) + re(random = ~1 | subject), family = JSU, data = na.omit(sample.500))

Fitting method: RS()

---------------------------------------------------------------------------------------------------------------------

Mu link function: identity

Mu Coefficients:

Estimate Std. Error t value Pr(>|t|)

(Intercept) 56.6967 0.64547 87.838 < 2e-16 ***

cs(activity) 0.43963 0.01351 32.542 < 2e-16 ***

cs(hour) 0.27568 0.03483 7.914 3.49e-15 ***

astron.seasonSpring 0.46370 0.66597 0.696 0.486307

astron.seasonSummer -1.95510 0.79473 -2.460 0.013948 *

astron.seasonFall -1.41688 0.85717 -1.653 0.098442 .

cs(Temperature) -0.04696 0.01214 -3.868 0.000112 ***

cs(hour):astron.seasonSpring 0.16001 0.04677 3.421 0.000632 ***

cs(hour):astron.seasonSummer 0.03834 0.05088 0.754 0.451163

cs(hour):astron.seasonFall 0.11059 0.06304 1.754 0.079490 .

---

Signif. codes: 0 ‘***’ 0.001 ‘**’ 0.01 ‘*’ 0.05 ‘.’ 0.1 ‘ ’ 1

---------------------------------------------------------------------------------------------------------------------

NOTE: Additive smoothing terms exist in the formulas:

i) Std. Error for smoothers are for the linear effect only.

ii) Std. Error for the linear terms maybe are not accurate.

---------------------------------------------------------------------------------------------------------------------

No. of observations in the fit: 3000

Degrees of Freedom for the fit: 26.96501

Residual Deg. of Freedom: 2973.035

at cycle: 17

Global Deviance: 20412.09

AIC: 20466.02

SBC: 20627.98

******************************************************************************


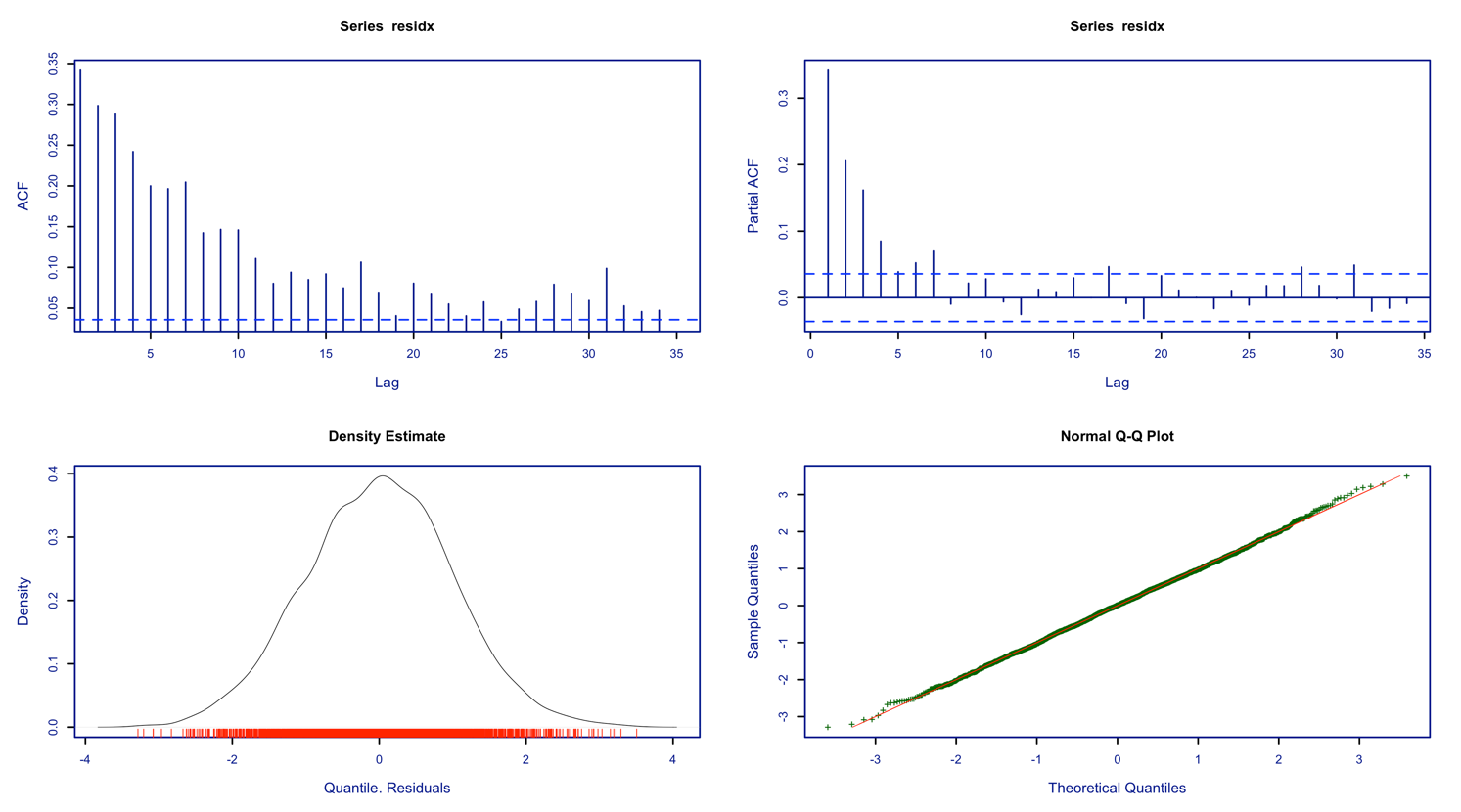


**FIGURE S4**. Diagnostic plot of a gamlss model fit with a random sample of 500 data points per subject (*hrm8.random*). ACF plots show reduction of autocorrelation of the data, with autocorrelation coefficients of lag 1 = 0.34 and lag 24 = 0.05.


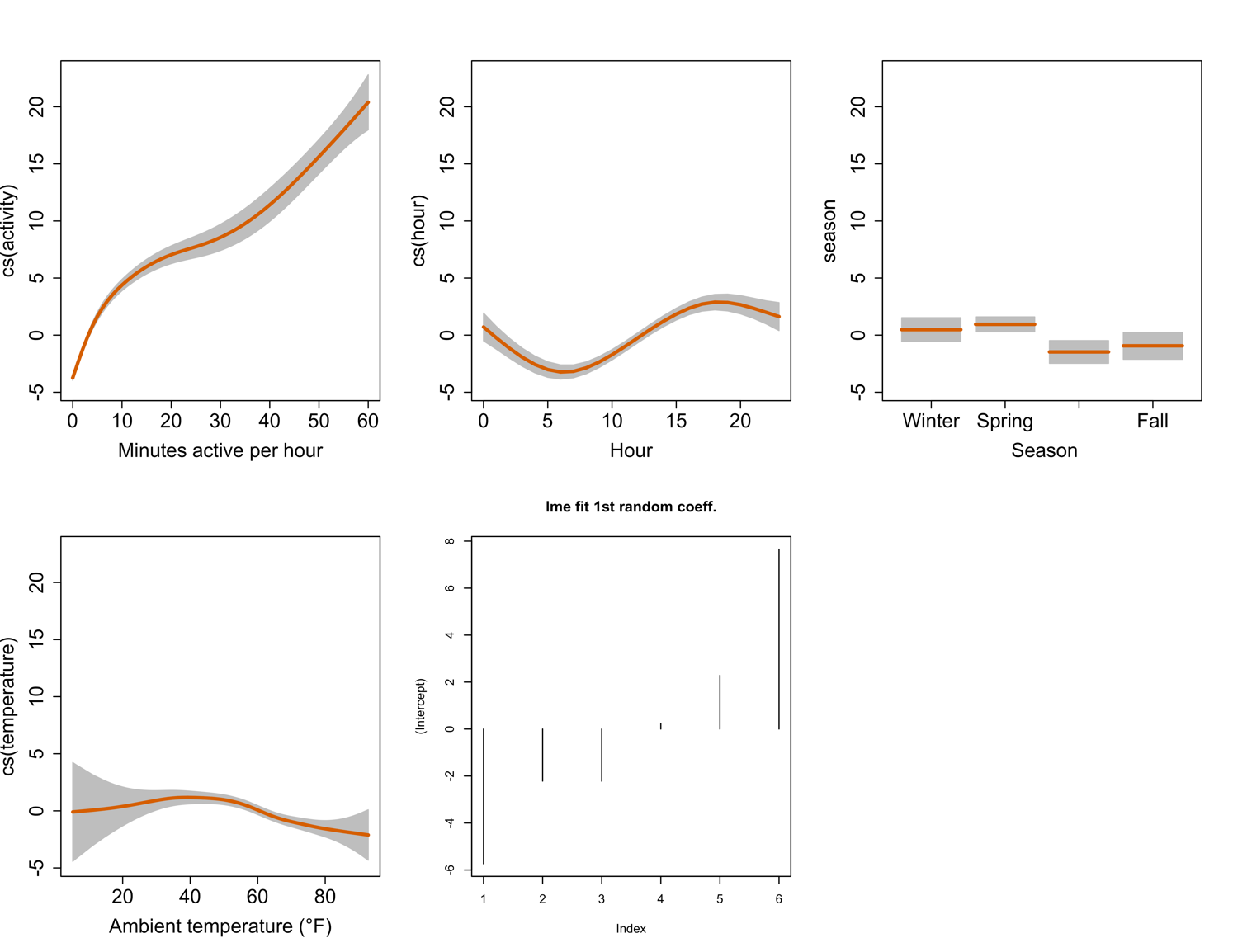


**FIGURE S5.** *Termplot* of the model *hrm8.random*, fit with a random subset of data (3000 data points). On the y-axis the partial effects of selected explanatory variables on the hourly average of heart rate (bpm) for six captive scimitar-horned oryx. Heart rate estimates are represented by the trend lines (cubic spline smooth predictors [cs] for activity, hour and ambient temperature and linear predictors for season). Estimates did not differ from the estimates obtained with the model fit with the full dataset (*hrm8*), but the gray shaded areas corresponding to the estimates’ standard errors are wider.

**Table S4.** Comparison of heart rate for scimitar-horned oryx with values published for other ungulate species. Data sorted by increasing body mass.

| **Species** | **Common name** | **Species weight range^1^**  **[kg]** | **Weight measured in study**  **[kg]** | **Heart rate [bpm]** | **Source** |
| --- | --- | --- | --- | --- | --- |
| *Capreolus capreolus* | Roe deer | 15-50 | — | 60.5-254.8 | (Theil et al., 2004) |
| *Capreolus capreolus* | Roe deer | 15-50 | — | 66-76 | (Reimoser, 2012) |
| *Oryx dammah* | Scimitar-horned oryx | 100-210 | 112-158 | 31-158 | This study |
| *Cervus elaphus* | Red deer/Elk | 75-340 | — | ~38-85 | (Arnold et al., 2004) |
| *Cervus elaphus* | Red deer/Elk | 75-340 | — | 44-71 | (Reimoser, 2012) |
| *Cervus elaphus* | Red deer/Elk | 75-340 | 135-155 | ~40-70 | (Turbill et al., 2011) |
| *Rangifer tarandus* | Reindeer | 60-318 | 42.8-61.3 | 40-103 | (Trondrud et al., 2021) |
| *Rangifer tarandus* | Reindeer | 60-318 | 65-87 | 34.2-58.5 | (Mesteig et al., 2000) |
| *Alces alces* | Moose | 200-825 | — | 40.5-71.9 | (Græsli et al., 2020) |
| *Taurotragus oryx* | Eland | 400-1,000 | — | ~38-111 | (Zizkova et al., 2013) |

^1^(Nowak and Walker, 1999)

**References**

Arnold, W., Ruf, T., Reimoser, S., Tataruch, F., Onderscheka, K., Schober, F., et al. (2004). Nocturnal hypometabolism as an overwintering strategy of red deer (*Cervus elaphus*). *Am. J. Physiol. Regul. Integr. Comp. Physiol.* 286, 174–181.

Græsli, A. R., Thiel, A., Fuchs, B., Singh, N. J., Stenbacka, F., Ericsson, G., et al. (2020). Seasonal Hypometabolism in Female Moose. *Front. Ecol. Evolv.* 8, 107.

Mesteig, K., Tyler, N. J., and Blix, A. S. (2000). Seasonal changes in heart rate and food intake in reindeer (*Rangifer tarandus tarandus*). *Acta Physiol. Scand.* 170, 145–151.

Nowak, R. M., and Walker, E. P. (1999). *Walker’s Mammals of the World*. JHU Press.

Reimoser (2012). “Influence of anthropogenic disturbances on activity, behavior and heart rate of roe deer (*Capreolus capreolus*) and red deer (*Cervus elaphus*), in context of their daily and yearly activity patterns,” in *Deer: Habitat, Behavior and Conservation*, ed. Cahler A A Marsten J (Nova Science Publishers, Inc.), 1–96.

Theil, P. K., Coutant, A. E., and Olesen, C. R. (2004). Seasonal changes and activity-dependent variation in heart rate of roe deer. *J. Mammal.* 85, 245–253.

Trondrud, L. M., Pigeon, G., Albon, S., Arnold, W., Evans, A. L., Irvine, R. J., et al. (2021). Determinants of heart rate in Svalbard reindeer reveal mechanisms of seasonal energy management. *Philos. Trans. R. Soc. Lond. B Biol. Sci.* 376, 20200215.

Turbill, C., Ruf, T., Mang, T., and Arnold, W. (2011). Regulation of heart rate and rumen temperature in red deer: Effects of season and food intake. *J. Exp. Biol.* 214, 963–970.

Zizkova, K., Kotrba, R., and Kocisova, A. (2013). Effect of changes in behaviour on the heart rate and its diurnal variation in a male and a female eland (*Taurotragus oryx*). *Agricultura tropica et subtropica* 46, 29–33.
